# Perioperative acute hypertension—role of Clevidipine butyrate

**DOI:** 10.3389/fphar.2014.00197

**Published:** 2014-08-27

**Authors:** Lakshmi N. Kurnutala, Suren Soghomonyan, Sergio D. Bergese

**Affiliations:** ^1^Department of Anesthesiology, Henry Ford Hospital, Wayne State UniversityDetroit, MI, USA; ^2^Department of Anesthesiology, Wexner Medical Center, Ohio State UniversityColumbus, OH, USA; ^3^Department of Neurological Surgery, Wexner Medical Center, Ohio State UniversityColumbus, OH, USA

**Keywords:** perioperative period, arterial hypertension, anti-hypertensive medications, calcium channel blockers, dihydropyridine, Clevidipine

Arterial hypertension (AH) is one of the most common pathological conditions affecting the general population which contributes to pathogenesis of various diseases and worsens the treatment outcome (James et al., 2014). It increases the perioperative morbidity and mortality, frequently result in cancelation of surgical procedures and increases treatment-associated costs (Handler, 2006). Perioperative hypertension may occur in patients with pre-existing arterial hypertension or manifest as a de novo phenomenon (Vuylsteke et al., 2000; Varon and Marik, 2008).

Induction of general anesthesia is related with significant stress and sympathetic over-activation. The systolic blood pressure (SBP) in normotensive patients may increase by up to 20–30 mm Hg, while hypertensive patients may have an exaggerated reaction—SBP in these patients may increase up to 90 mm Hg (Ahuja and Charap, 2010). On the other hand, anesthesia-induced sympathetic suppression with diminished baroreceptor reflex takes place during the maintenance phase which frequently causes sustained arterial hypotension. Postoperatively, these patients present with labile BP, frequent and rebound hypertension, and have an increased risk of postoperative complications (Goldman and Caldera, 1979; Wolfsthal, 1993).

Joint National Committee on Prevention, Detection, Evaluation, and Treatment of High Blood Pressure (JNC-7 and JNC-8) classifications and guidelines are currently applicable to perioperative patients as well (James et al., 2014; Chobanian et al., 2003).

According to JNC-7 guidelines for evaluation and treatment of hypertensive emergencies, an immediate intervention is required in cases of hypertensive emergencies to reduce the SBP by 10–15% (no more than 25%) within the first hour. Reduction of the absolute BP to 160/110 mmHg should be done gradually over the following 2–6 h (Aggarwal and Khan, 2006; Flanigan and Vitberg, 2006; Pollack and Varon, 2008; Varon, 2008; De Gaudio et al., 2009; Hays and Wilkerson, 2010; Kuppasani and Reddi, 2010; Smithburger et al., 2010; Polly et al., 2011). Only in patients with aortic dissection, the SBP should be reduced to less than 120 mmHg within 20 min. In hypertensive emergencies associated with ischemic stroke, BP must be decreased to less than 180/110 before thrombolytic therapy may be administered (Pollack and Varon, 2008; Varon, 2008; De Gaudio et al., 2009; Hays and Wilkerson, 2010; Polly et al., 2011). Abrupt BP reduction should be avoided as it can result in critical blood flow reduction and ischemic end organ damage (Varon, 2008; De Gaudio et al., 2009; Smithburger et al., 2010; Polly et al., 2011).

Since overshooting a target BP in hypertensive patients is associated with worse outcome, many treatment protocols require invasive arterial blood pressure monitoring during anesthesia in high risk cases (Pollack and Varon, 2008; Rhoney and Peacock, 2009).

Hypertensive emergencies should be treated aggressively, using quick-onset intravenous medications, whereas hypertensive urgencies do not always require such aggressive treatment. Longer acting oral medications (labetalol, clonidine, etc.) may be more appropriate in situations of hypertensive urgency. However, caution should be exercised when using anti-hypertensive agents in the acute setting. An overly aggressive treatment approach may lead to organ hypoperfusion (Rodriguez et al., 2010). Once the immediate threat of organ damage is diminished, BP should be gradually brought to baseline level within a period of 24–48 h (Peacock et al., 2009).

Characteristics of an ideal intravenous hypertensive agent are rapid onset and offset of action, low risk of hypotension, minimal drug interaction with other medications, lack of adverse reactions, wide therapeutic window, ease of titration, preservation of renal and hepatic functions, selectivity, low cost, possibility of easy transition to oral therapy, predictable response, and lack of effects on intracranial pressure (Levy, 1999; Oparil et al., 1999). Multiple intravenous medications are currently used to control the BP in the perioperative period, and all these medications have specific advantages and limitations (Kurnutala et al., 2013).

Selection of an optimal antihypertensive therapy for the perioperative period depends on the patient's individual characteristics and locally adopted guidelines (Pollack and Varon, 2008; Belsha, 2013).

*Clevidipine butyrate* is a Ca^2+^-channel antagonist that acts on the L-type Ca^2+^ channels regulating the influx of Ca^2+^ ions into the arteriolar smooth muscle cells during depolarization. It has recently raised significant interest among anesthesiologists and surgeons as a valuable agent to control AH during surgery because of its fast and selective action and ease of BP control. Clevidipine selectively dilates the arterioles and reduces peripheral resistance, thus increasing stroke volume and cardiac output. Like nicardipine and nifedipine, it belongs to the subclass of dihydropyridines—a group of drugs considered a first-line treatment for hypertensive emergencies because of strong vasodilating effects and low propensity to cause cardiac conduction and contractility abnormalities (Eisenberg et al., 2004; Nordlander et al., 2004; FDA/CDER, 2008; Bergese and Puente, 2010; The Medicines Company, 2011; Tulman et al., 2012).

Clevidipine butyrate was approved by FDA in 2008. Interestingly, it was the only intravenous antihypertensive drug approved during the past 10 years.

The pharmacologic characteristics of clevidipine are beneficial for its use during the perioperative period. The drug formulation represents a racemic mixture of equipotent S- and R-enantiomers (Noviawaty et al., 2008; Sorbera and Castanera, 2004). The preparation is a sterile lipid emulsion with pH of 6.0–8.0 ready for intravenous use (Sorbera and Castanera, 2004; Noviawaty et al., 2008). As with other lipid solutions (intralipid, propofol), strict asepsis must be maintained during administration to prevent bacterial contamination and growth. Clevidipine must be discarded 12 h after puncturing the stopper. The drug is metabolized by plasma and tissue esterases to inactive metabolites with a mean blood clearance rate of 0.121 lit. min^−1^ kg^−1^ (Ericsson et al., 1999a). Up to 99.7% of circulating clevidipine is bound to plasma proteins. The elimination rate does not depend on hepatic and renal clearance and it can be safely administered in patients with hepatic and renal pathology (Ericsson et al., 1999b).

During anesthesia, clevidipine is administered as a slow rate intravenous infusion. As with other lipid solutions, the drug is contraindicated in cases of allergy to soy and egg products and in patients with defective lipid metabolism. The drop in BP equaling 4–5% of the initial value takes place within 2–4 min after starting the infusion.

Clevidipine has many features of an ideal antihypertensive drug that can be used in emergency situations. These features include fast onset and offset of clinical effects, ease of titration, small volume of distribution and fast elimination independent on hepatic and renal clearance (Ericsson et al., 1999a,b; Prlesi and Cheng-Lai, 2009).

Clinical trials showed that the racemic formulation of clevidipine is superior to L or R enantiomers in controlling AH (Schwieler et al., 1999; Ericsson et al., 2000).

In patients undergone elective cardiac interventions, clevidipine was effective in controlling the mean BP and systemic vascular resistance in a dose-dependent manner. The drug did not cause any cardiovascular instability or metabolic changes (Kieler-Jensen et al., 2000; Ericsson et al., 2001; Bailey et al., 2002).

Two randomized double blind, placebo controlled trials performed in cardiosurgical patients (ESCAPE1 and ESCAPE2) showed that in 92.5–91.8% of patients the desired SBP values could be achieved within 4–7 min after starting the drug infusion (Levy et al., 2007; Singla et al., 2008). Aronson et al., compared clevidipine with other commonly used antihypertensive drugs in cardiac surgery (Aronson et al., 2008). In this study, the clevidipine group achieved superior BP control compared with other drugs and had a significantly lower 30-day mortality rate than sodium nitroprusside. Pollack et al. (2009) evaluated the efficacy and safety of prolonged infusion of clevidipine (up to 96 h) (Pollack et al., 2009). In 88.9% of patients, the target SBP could be achieved within half an hour after starting the infusion. In 1.6% of cases the SBP dropped below the target range.

The ACCELERATE trial evaluated the efficacy of clevidipine in management of severe AH associated with intracerebral hemorrhage (Graffagnino et al., 2013). Again, the clevidipine infusion helped to rapidly reduce the BP to the desired level (<160 mm Hg) within a few minutes.

Clevidipine can be effectively used during elective neurosurgical procedures, including neurovascular interventions, to control the BP during intraoperatively and in the postoperative period (Bekker et al., 2010; Varelas et al., 2014).

Another area of surgery, where clevidipine can be used with success is surgical resection of pheochromocytoma (Kline, 2010; Bettesworth et al., 2013).

More research is required to clarify the safety of clevidipine in pregnant and breastfeeding patients. In these patients, the drug should be used with caution and only when the benefits of such therapy will clearly outweigh the potential risks (The Medicines Company, 2011). Another area of research is pediatric population, even though several case reports suggest that clevidipine may be considered a safe option in pediatric patients (Tobias et al., 2009, 2011, 2013; Towe and Tobias, 2010; Tobias and Hoernschemeyer, 2011).

As a conclusion, Clevidipine butyrate is an easily controlled and effective drug that can be safely used in various surgical patients to control the BP. The drug has minimal side effects and can be titrated to reach the desired clinical effect. It can be safely used in patients with hepato-renal pathology. Further research is required to clarify clevidipine's efficacy, safety and limitations in pregnant and breastfeeding women and pediatric surgical patients.

## Conflict of interest statement

The authors declare that the research was conducted in the absence of any commercial or financial relationships that could be construed as a potential conflict of interest.
